# PathoSeq-QC: a decision support bioinformatics workflow for robust genomic surveillance

**DOI:** 10.1093/bioinformatics/btaf102

**Published:** 2025-03-07

**Authors:** Gabriele Leoni, Mauro Petrillo, Victoria Ruiz-Serra, Maddalena Querci, Sandra Coecke, Tobias Wiesenthal

**Affiliations:** European Commission, Joint Research Centre (JRC), Ispra, 21027, Italy; Seidor Italy S.r.l., Milan, 20122, Italy; European Commission, Joint Research Centre (JRC), Geel, 2440, Belgium; European Commission, Joint Research Centre (JRC), Ispra, 21027, Italy; European Commission, Joint Research Centre (JRC), Ispra, 21027, Italy; European Commission, Joint Research Centre (JRC), Geel, 2440, Belgium

## Abstract

**Motivation:**

Recommendations on the use of genomics for pathogens surveillance are evidence that high-throughput genomic sequencing plays a key role to fight global health threats. Coupled with bioinformatics and other data types (e.g., epidemiological information), genomics is used to obtain knowledge on health pathogenic threats and insights on their evolution, to monitor pathogens spread, and to evaluate the effectiveness of countermeasures. From a decision-making policy perspective, it is essential to ensure the entire process’s quality before relying on analysis results as evidence. Available workflows usually offer quality assessment tools that are primarily focused on the quality of raw NGS reads but often struggle to keep pace with new technologies and threats, and fail to provide a robust consensus on results, necessitating manual evaluation of multiple tool outputs.

**Results:**

We present PathoSeq-QC, a bioinformatics decision support workflow developed to improve the trustworthiness of genomic surveillance analyses and conclusions. Designed for SARS-CoV-2, it is suitable for any viral threat. In the specific case of SARS-CoV-2, PathoSeq-QC: (i) evaluates the quality of the raw data; (ii) assesses whether the analysed sample is composed by single or multiple lineages; (iii) produces robust variant calling results via multi-tool comparison; (iv) reports whether the produced data are in support of a recombinant virus, a novel or an already known lineage. The tool is modular, which will allow easy functionalities extension.

**Availability and implementation:**

PathoSeq-QC is a command-line tool written in Python and R. The code is available at https://code.europa.eu/dighealth/pathoseq-qc.

## 1 Introduction

Technology development in the last decades has allowed the extensive production of omics biology data (Dai and Shen 2022). This has played a crucial role in the rapid advancement of comprehensive analysis and identification of complex biological patterns. The importance of next-generation sequencing (NGS) and genomics became particularly manifest during the coronavirus disease 2019 (COVID-19) pandemic to provide evidence in support to decision making and vaccine and treatment (Cen *et al.* 2023). NGS was instrumental to quickly obtain raw genomic sequences from SARS-CoV-2, the causing disease virus, which in turn were processed by bioinformatics tools to reconstruct (i.e., assembly) the final sequence of the virus (Wu *et al.* 2020, Zhou *et al.* 2020, Mercer and Salit 2021). Eventually, the global sharing of these genomic data marked a crucial milestone in combating the pandemic (Harrison *et al.* 2021, Khare *et al.* 2021).

One of the most concerning risks in case of urgent need of rapid production and sharing of genomic data is that quality of the data might be not always guaranteed (WHO 2022), or that rigorous quality assessments are often not consistently implemented or, if so, they are not publicly available (Jacot *et al.* 2021). This lack of assurance hinders the trustworthiness and comparison of downstream analyses (Zufan *et al.* 2023). For example, in the case of SARS-CoV-2, consensus of lineages genomic sequences and variants of concern (VOCs) are defined according to the presence of nucleotide mutations identified, via a variant calling process, with respect to the Wuhan reference sequence. In addition, identified mutations and VOCs can be used by epidemiological modelling to understand the spread of infectious disease through populations. Moreover, this variant calling process depends on the chosen bioinformatics software and corresponding parameters (Garcia-Prieto *et al.* 2022), and the quality of the input raw genomic data. Consequently, in a decision-making context based on scientific evidence, such as policymaking and public health, it is of upmost importance to accurately assess and share the quality of the used genomic data for genomic surveillance of pathogens with pandemic and epidemic potential (WHO 2023).

It exists a wide array of tools specifically designed to assess the quality of genomic raw reads [for a representative list see (Expósito *et al.* 2020)]. Additionally, many attempts have been made to create SARS-CoV-2 analyses pipelines that incorporate raw reads quality control (QC) evaluations, such as the widely used per-base sequence quality, per-sequence quality score and sequence length distribution (Oliveira *et al.* 2022, Jalal *et al.* 2023). However, while these tools efficiently determine the quality of raw reads data, only a subset of workflows investigate the quality of the pipeline’s results and evaluate the sample’s genomic homogeneity, which might strongly influence critical analyses steps, such as variant calling and, therefore, lineage assignment. Moreover, pipelines usually focus on lineage assignment only and do not extend their functionalities to additional analyses, such as the identification of subgenomic RNAs (sgRNAs), which are hypothesized to provide indications of active viral replication (Chen *et al.* 2022). On the other hand, tools able to perform specific analyses, often do not implement sufficient raw data QC evaluation, such as in the case of the *Periscope* tool for sgRNA identification (Parker *et al.* 2021). Actually, raw data QC is a critical responsibility of the researcher and should be conducted during the initial stages prior to analysis with these specific bioinformatics tools. On top of the limitations mentioned, there is a pressing need for multifunctional integrated tools that can accommodate emerging technology requirements and novel threats, rather than relying on the current practice of evaluating multiple tool outputs to reach a consensus on results, highlighting the need for more streamlined and integrated approaches.

To address the limitations and needs outlined above, we here introduce PathoSeq-QC, a command-line bioinformatics pipeline developed in Python and R, structured as a sequential workflow, that comprehensively analyses raw NGS data in FASTQ format. PathoSeq-QC is highly customizable, leveraging up to 15 different tools, including raw reads QC evaluation, reads filtering, reads mapping, BAM preprocessing, variant calling, variant annotation and lineage assignment, among others, facilitating the analysis of pathogenic virus NGS data. Although conceptualized and crafted during the pandemic for SARS-CoV-2, the pipeline is versatile and adaptable to other viral pathogens, much like a living document that evolves with new knowledge. It has been successfully tested on A(H5N1) and Oropouche viruses. Additionally, it is particularly suitable for large-scale data analysis due to its multithreaded architecture and optimized use of computational resources. PathoSeq-QC is accessible at https://code.europa.eu/dighealth/pathoseq-qc.

## 2 Methods

### 2.1 PathoSeq-QC strategy

PathoSeq-QC workflow takes as input NGS raw data from samples in which viral pathogens are expected to be present, such as from clinical samples. Supplementary Figure S1 provides the list of steps with the corresponding tools implemented in PathoSeq-QC. Each analysis is a separate module, making it easy to add new functionalities by simply adding other modules. Originally developed for SARS-CoV-2 PathoSeq-QC can also be used with other viral pathogens. For example, we successfully tested it on A(H5N1) (run id: SRR29851702) and Oropouche (run id: SRR14711849) viruses (see Supplementary Fig. S6).

PathoSeq-QC is designed to perform three main tasks on NGS raw data from viral pathogens sequenced with Illumina paired-end technology (either via shotgun or via amplicon-based methods). These three tasks are:

1. *Evaluation of raw data quality.* PathoSeq-QC assesses the overall quality of raw sequencing data, including sequence coverage, depth, and quality issues (Petrackova *et al.* 2019) that may affect downstream analyses. It uses tools like *fastp* (Chen *et al.* 2018), *samtools depth*, and *samtools coverage* (Danecek *et al.* 2021) for this purpose. Additionally, PathoSeq-QC corrects for sequencing artefacts using the GATK best practice workflow, including mapping data recalibration and deduplication (DePristo *et al.* 2011).
2. *Analysis of genomic homogeneity.* PathoSeq-QC evaluates the genomic homogeneity of the analysed sample. This is crucial, as any detected heterogeneity can be indicative of contamination and/or co-infections in human samples or, in the case of wastewater samples, a deliberate mixture.
3. *Designation and classification.* Pathogen lineage nomenclature, or the designation of epidemiologically distinct groups below the level of species, is essential for effective research, treatment, and communication about diseases. To ensure robust lineage designation, PathoSeq-QC implements three variant callers—*GATK HaplotypeCaller* (Poplin *et al.* 2017), *LoFreq* (Wilm *et al.* 2012) and *iVar* (Grubaugh *et al.* 2019) embedded in *Freyja* (Karthikeyan *et al.* 2022)—to increase the confidence of identified mutations.

In the case of SARS-CoV-2, the *Freyja* tool is used in both steps 2 and 3, while *Pangolin* (O’Toole *et al.* 2021) and *Virstrain* (Liao *et al.* 2022) are implemented to assesses whether the viral pathogen can be confidently assigned to a single known subtype (lineage or clade) in step 3. Additionally, a custom procedure is implemented to identify novel (i.e. not yet classified) and recombinant SARS-CoV-2 lineages generated *in-silico* (described in Supplementary Section S2). For A(H5N1) virus, LABEL (https://wonder.cdc.gov/amd/flu/label/), and GenoFlu (Youk *et al.* 2023) are used in step 3. PathoSeq-QC offers also a suite of analyses, including subgenomic RNAs (sgRNAs) discovery, variant calling and variant annotation.

### 2.2 Installation and dependencies

PathoSeq-QC is written in Python v3.7 and R v4.2 and is designed for Unix-like operating systems (OS) (tested on Ubuntu-20.04.4 LTS). It leverages multithreading and is compatible with High Performance Computing (HPC) infrastructures [tested on the Joint Research Centre Big Data Analytics Platform—BDAP—infrastructure based on HTCondor (Erickson *et al.* 2018)].

Dependencies and software are managed with *conda* and installed on separate environments to ensure precise version tracking, reproducibility, and prevent dependency issues or software incompatibilities. Additionally, this approach allows for high modularization of the tool as presented in Supplementary Fig. S1. Installation is automated via a script, and full instructions on how to install and run PathoSeq-QC, explanation of input and output formats as well as examples are provided in the README file included in the repository.

### 2.3 Performances and flexibility

The details of PathoSeq-QC flexibility, performances, and computational utilization and efficiency are provided as Supplementary Material (Supplementary Sections S3–S6).

### 2.4 Use case dataset

Raw NGS reads data belonging to the representative SARS-CoV-2 sequences were obtained from the COVID-19 data portal (https://www.covid19dataportal.org/search/sequences? crossReferencesOption=all&overrideDefaultDomain=true&db=representative-sequences&size=15).

## 3 Results

### 3.1 Data flexibility

PathoSeq-QC was tested on shotgun and amplicon *in-silico* datasets to assess its performances. The details of testing are provided as Supplementary Material (Supplementary Section S4). Overall, our tool showed good performances scores (mean values of precision: 0.91, accuracy: 0.85, recall: 0.78, Fscore: 0.86 for shotgun *in-silico* NGS data). We also benchmarked PathoSeq-QC against V-pipe 3.0 (Fuhrmann *et al.* 2024), one of the most recently published computational pipeline designed for analysing NGS data of short viral genomes, using high-coverage (>1000x) NGS data. Our tool showed slightly higher performance compared to V-pipe 3.0, though the difference was not statistically significant (Supplementary Fig. S5). Importantly, PathoSeq-QC includes downstream lineage designation steps, a feature not available in V-pipe 3.0, making it a more comprehensive alternative for SARS-CoV-2 analyses. Versatility has been successfully verified by running PathoSeq-QC on raw data from viral isolates genomes of A(H5N1) and Oropouche viruses. Examples of output files on these viral species are provided in the Supplementary Section S6. A detailed guide on how to interpret the output files can be found in the README file in the PathoSeq-QC repository. The datasets used to evaluate the performances are all freely available (DOIs in the Supplementary Material).

### 3.2 Computational performance evaluation

We tested the computational performance of PathoSeq-QC in two scenarios: using a relatively small dataset and a larger one. For the first test, we first generated a small *in-silico* dataset consisting of 40 000 paired-end reads (in FASTQ format) using a true recombinant sequence (SARS-CoV-2 XD lineage). In a Desktop Linux environment and with 4 CPUs (see Supplementary Section S3), PathoSeq-QC required a maximum of 3 GB of RAM memory and was able to perform all non-optional modules and to correctly assign the test dataset to the XD lineage (e.g. QC, variant calling and lineage assignment steps) in 5 min (Supplementary Fig. S3). For the second test, which involved also the *bbmap* and *Freyja* optional steps, we used a bigger dataset consisting of 472 109 paired-end reads. This time, PathoSeq-QC took approximately 14 min using the Linux machine as before and a total of 10 CPUs.

### 3.3 Use case

We evaluated PathoSeq-QC’s ability to identify quality deficiencies in publicly available SARS-CoV-2 datasets, specifically the COVID-19 Data Portal (Harrison *et al.* 2021). We analysed the representative lineage sequences dataset (see Section 2). Out of 224 samples, only 86 samples had available raw data (in FASTQ format). Although reads quality was not an issue, 38 samples (44%) had low mean sequence coverage (<20x) and 39 samples (45%) showed signs of high genomic heterogeneity (Supplementary Figs S7 and S8). Since genomic heterogeneity might strongly affect variant calling results, we manually inspected the PathoSeq-QC’s variant calls of three randomly selected samples (run ids: ERR6136423, ERR6187498, ERR7541889) and compared them with their corresponding assembled sequences on the COVID-19 data portal. We observed nine variants with low allele frequencies (Supplementary Table S1), indicating high genomic heterogeneity (Boscolo Bielo *et al.* 2023). Additionally, five variants were identified by *iVar* but not by *GATK* and *LowFreq* tools (Supplementary Table S2). To determine whether this was due to sensitivity limitations of the tools or potential errors in the assembled sequence, we manually inspected the five variants in question. Variant 21987: G-A in samples with run ids: ERR6136423 and ERR6187498 failed *iVar’*s internal QCs, suggesting that it may be a technical and/or assembly error. The other four variants (8835: T-C and 25350: C-T for run id: ERR7541889, 24410: G-A for run id: ERR6187498 and 28461: A-G for run id: ERR618798) had low AF values (≤0.5) according to *iVar* results, supporting the hypothesis of high heterogeneity in this sample. Moreover, *iVar* and *GATK* detected variant 29742: G-T, which is present in many VOCs, but was poorly supported by robust reads evidence (i.e. low reads count). Overall, these results highlight the importance of robust quality assessments in variant calling and lineage assignment.

### 3.4 Considerations on deduplication

PathoSeq-QC includes a deduplication step, a bioinformatics procedure that removes reads likely originating from the same genetic fragment during sequencing (e.g., due to PCR amplification artefacts). Deduplication can significantly influence variant calling by different read support counts, ultimately affecting the final set of variants on lineages sequences. To illustrate this, we manually inspected the mapping data of a single sample (run id: ERR7541889) prior and after deduplication. We focused on variant 29742: G-T, that was previously detected with insufficient read support. Without deduplication, we observed 171 supporting reads, but only two remained after deduplication (1.17%), negatively impairing the quality of the called variant (Supplementary Fig. S9).

## 4 Conclusions

Genomics can provide new insights into health pathogenic threats such as viruses, their evolution (Markov *et al.* 2023), and their spread. Additionally, genomics can inform decision-making on countermeasures to control outbreaks. However, there is a current need for a unified, best-practices approach to bioinformatics among laboratories working on a common outbreak problem (Foster *et al.* 2022). This approach also requires publicly available and standardized benchmark data to support accurate and timely outbreak investigation and surveillance (Xiaoli *et al.* 2022). Recently, Connor and colleagues issued a series of relevant recommendations on this topic (Connor *et al.* 2024). PathoSeq-QC represents a first attempt to implement these recommendations, fortifying high-throughput genomic sequencing as an integral component of routine pathogen surveillance. By establishing quality criteria for the global sharing of data and results, PathoSeq-QC maintains data integrity to mitigate the risk of erroneous conclusions that could influence decision-making in critical scenarios.

Our assessment of PathoSeq-QC performance, highlights the importance of quality evaluation at different analyses steps to prevent the discovery of erroneous nucleotide mutations and ensure accurate final genomic sequences (Supplementary Fig. S9). For example, we identified samples with low genomic homogeneity, which might strongly impact downstream analyses. These samples may have been contaminated during sampling or preprocessing of clinical samples, making our findings particularly relevant given the dataset’s intended use as a reference for SARS-CoV-2 lineages.

PathoSeq-QC's adaptable architecture represents a step forward in elevating the adoption of reliable and robust bioinformatics pipeline for effective pathogen genomic surveillance on a global scale. An added value of PathoSeq-QC is its ability to handle NGS raw data from complex matrices, such as wastewater samples (manuscript in preparation). Due to its modular design, we envision smooth future implementation of this new feature in PathoSeq-QC’s workflow. Furthermore, our next steps will also focus on integrating the use of raw data from emerging sequencing technologies (e.g., nanopore) and from various pathogens, including novel health threats, for which we will leverage our work with the A(H5N1) and Oropouche viruses.

## Supplementary Material

btaf102_Supplementary_Data

## Data Availability

The data underlying this article are available in the article and in its online supplementary material.
