# Supplementary material for: PathoSeq-QC: a decision support bioinformatics workflow for robust genomic surveillance
Source: Bioinformatics. 2025 Mar 7;41(4):btaf102. doi: 10.1093/bioinformatics/btaf102 (PMC11961196; doi:10.1093/bioinformatics/btaf102)
Supplement: btaf102_Supplementary_Data [file btaf102_supplementary_data.pdf]

SUPPLEMENTARY MATERIAL

Contents

S.1 PathoSeq-QC workflow scheme 2
S.2 Custom procedures of PathoSeq-QC 3
S.3 PathoSeq-QC memory usage test 5
S.4 PathoSeq-QC performance on *in-silico* shotgun and amplicon NGS raw reads 6
S.5 PathoSeq-QC comparison with V-pipe 7
S.6 PathoSeq-QC species flexibility 8
S.7 Coverage analyses on the representative sequence dataset 9
S.8 Heterogeneity analyses 10
S.9 Impact of Deduplication on Variant Support for raw data run ID: ERR7541889 11
S.10 Supplementary tables 12
References 14

S.1 PathoSeq-QC workflow scheme

PathoSeq-QC is a highly customisable workflow (summarised in Fig. S1) of generic and pathogen-specific tools aimed to improve the trustworthiness of genomic surveillance analyses and conclusions.

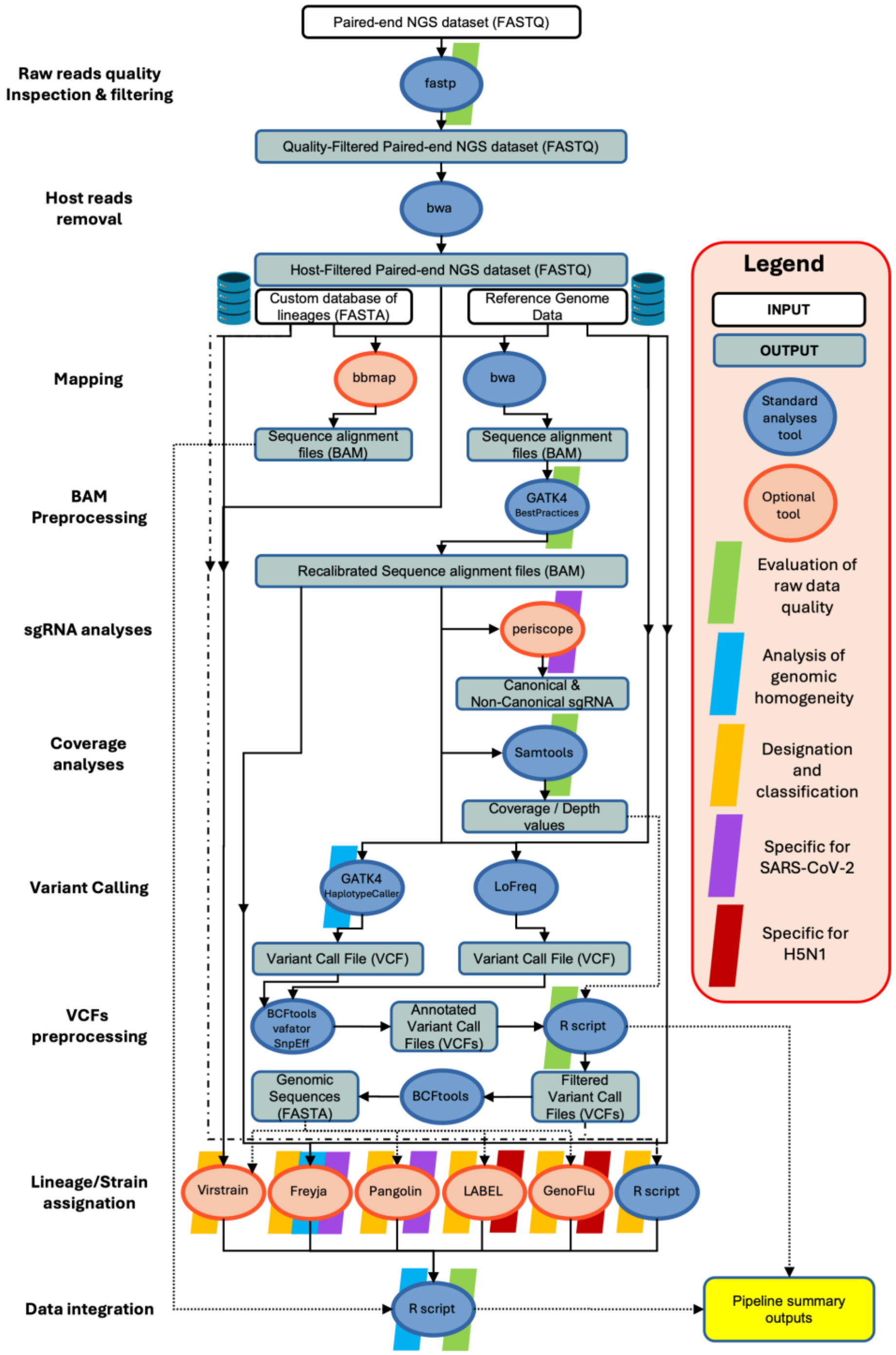

Figure S1. PathoSeq-QC workflow scheme. The list of the different steps employed by the pipeline is provided on the left part of the scheme. The tools used by the main and optional modules are embedded in blue and orange ovals, respectively.

S.2 Custom procedures of PathoSeq-QC

PathoSeq-QC can implement custom procedures tailored to specific methodologies. As proof, we have implemented two custom procedures aimed to assign a lineage to a sample.

The first procedure is meant to identify the most probable lineage in samples where it is expected to be highly abundant (*e.g.*, clinical samples). It involves multiple steps based on the comparison of two sets of variants: *Set A*, represented by variants detected in a sample using GATK *HaplotypeCaller*, and *Set B*, comprising variants identified in a single reference lineage sequence. An explanatory example is shown in Supplementary Fig. S2.1 with SARS-CoV-2 lineages AY.3, AY.43.4, AY.5.3, and AY.102. Initially, the approach computes the *'A ∩ B x 100 / A'* values (the *'A ∩ B x 100 / A* ‘column in the table of Supplementary Fig. S2.1), representing the percentage of lineage variants observed in the sample relative to all set A variants. This percentage is calculated for all the lineages stored in the fully customizable database. Then, all percentages are ranked in descending order and corresponding lineages are assigned to increasing indices (the 'A ∩ B index' column). Similarly, *'A ∩ B x 100 / B'* values are calculated (*'A ∩ B x 100 / B* ‘column) as percentages of lineage variants relative to the total set B variants, then percentages are ranked in descending order and corresponding lineages are each assigned to an increasing second index ('B ∩ A index' column). The sum of the two indices constitutes the final 'Score' of each lineage (‘Score’ column). Lineages are sorted based on the 'Score', with the most probable lineage having the lowest assigned score.

The second procedure is aimed to help user in assessing whether a given sample will contain novel or recombinant SARS-CoV-2 lineages by using the percentages, indices and scoring calculated as described. We utilised two datasets generated in-silico, each comprising 100 random novel lineages (green points in Supplementary Fig. S2.2) and 100 recombinant lineages (blue points), to evaluate the efficacy of our custom approach in distinguishing between highly abundant novel and recombinant lineages of SARS-CoV-2. The novel lineages were crafted by randomly integrating known SARS-CoV-2 variants into its reference genome. Meanwhile, the recombinant lineages were constructed by randomly introducing a single breakpoint into the reference genome sequences of two distinct SARS-CoV-2 lineages and subsequently merging the resultant fragments. This custom approach was successful in highlighting differences between the two groups of samples by relying on the final Score and the sum of percentages that are generated as output. Two homogeneous lineages (AY.3 and BA.2) were also analysed (red points) for comparison.

It is important to underline that, although specifically designed for samples with one expected highly abundant SARS-CoV-2 lineage, additional studies are needed to clarify the sensibility of the here presented custom approaches, the possibility to apply them on samples with mix of lineages with comparatively lower abundances, such as wastewater samples, and the approach flexibility towards different species.

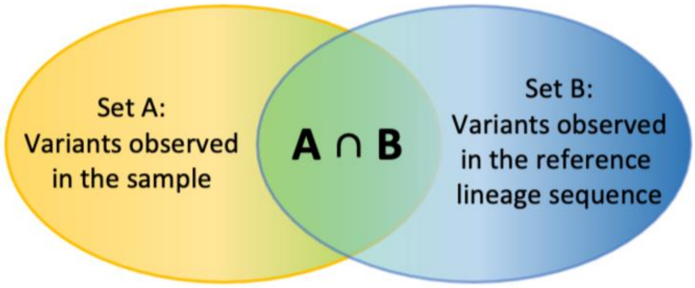

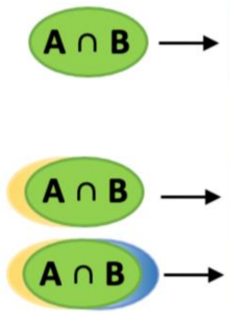

Lineages	A ∩ B x 100/A	A∩B index	A ∩ B x 100/B	B∩A index	Score
AY.3	100	1	100	1	2
AY.43.4	91.892	2	100	1	3
AY.5.3	91.892	2	100	1	3
AY.102	91.892	2	97.143	2	4

Supplementary Figure S2.1: Custom lineage assignment methodology.

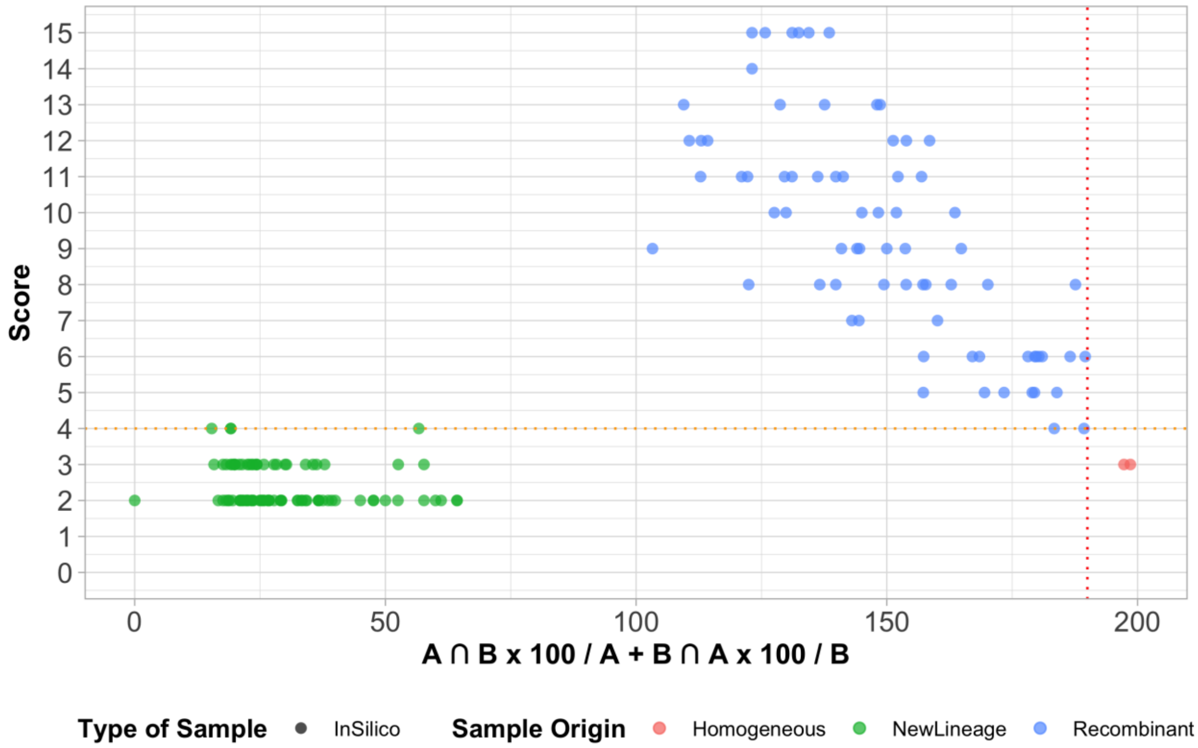

Supplementary Figure S2.2: PathoSeq-QC discrimination of novel and recombinant lineages. X axis is the algebraically sum of the percentage of lineage variants observed in the sample relative to all set A variants (*'A ∩ B x 100 / A'* values) and the percentages of lineage variants relative to the total set B variants (*'A ∩ B x 100 / B'* values). Y axis reports the 'Score' value, which is the algebraically sum of the 'A ∩ B index' and the 'B ∩ A index' columns showed in Figure S2.1.

S.3 PathoSeq-QC memory usage test

The analysis of PathoSeq-QC resource utilisation and efficiency was conducted by employing Python's 'memory profiler' module (https://pypi.org/project/memory-profiler/) on a 16 CPUs core Unix machine with Ubuntu 20.04.4 LTS operative system and 36 Gb of available memory. We used a custom dataset generated on February 2022 by randomly selecting 40,000 reads from the GISAID EPI_ISL_9879436-9879437 isolates (raw data for these samples are currently not available on GISAID). The dataset size was chosen in this way to ensure its reproducibility and usability. As shown in the Supplementary Fig. S3, the test dataset was processed in less than 5 minutes requiring a maximum of ~3 Gb of RAM memory during the mapping step, which occurs during the initial phase of the run. A second test was performed on the same machine setup with raw data from run id SRR34803899, which consists of 472,109 reads. The test took approximately 14 minutes and gave very similar results, in terms of memory consumption, with respect to the first test (data not shown).

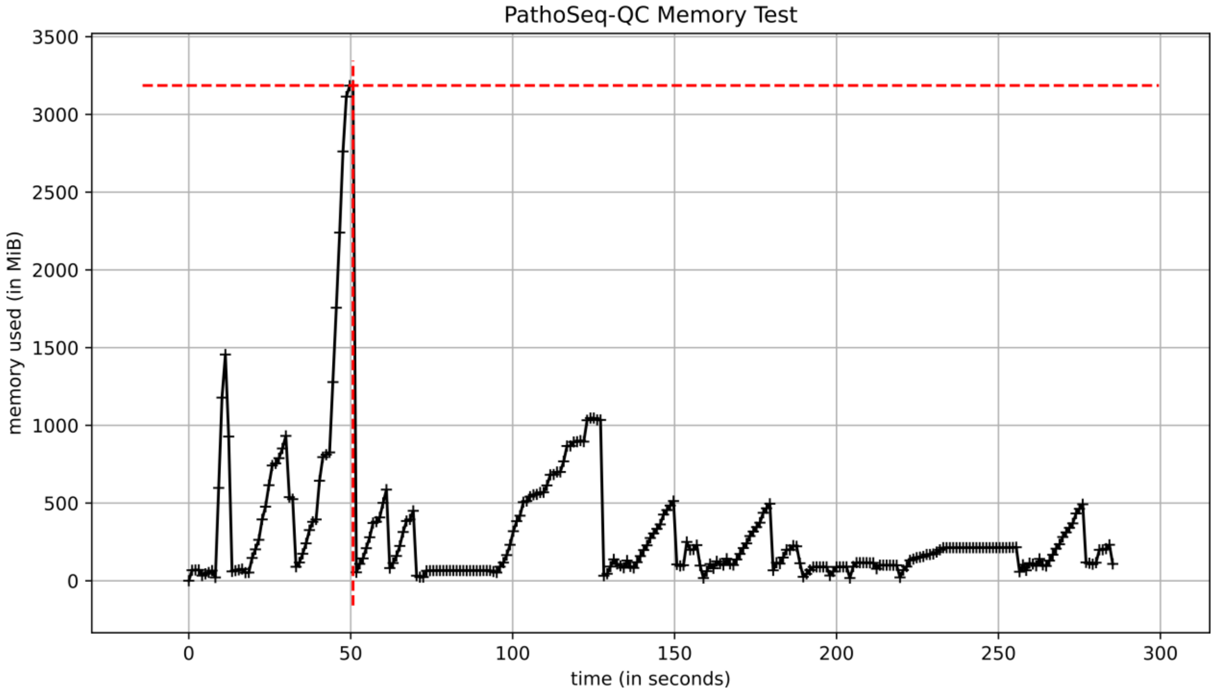

Supplementary Figure S3: PathoSeq-QC memory test. Generated by the Python's 'memory profiler' module (https://pypi.org/project/memory-profiler/).

S.4 PathoSeq-QC performance on *in-silico* shotgun and amplicon NGS raw reads

To evaluate PathoSeq-QC performance on reads from shotgun NGS experiments, we generated and used an *in-silico* shotgun NGS raw read dataset. This dataset, coupled with a full description of its composition, is available at the following DOI: 10.2905/f4cb0c69-5d43-4765-9ec8-e79cb28c21bc. To evaluate PathoSeq-QC performance on reads from NGS amplicon-based experiments, we generated and used an *in-silico* NGS amplicon-based raw read dataset. This dataset, coupled with a full description of its composition, is available at the following DOI: 10.2905/7914cd34-f80b-4145-9bc3-4ba04e337f14. Results are summarised in Supplementary Fig. S4.

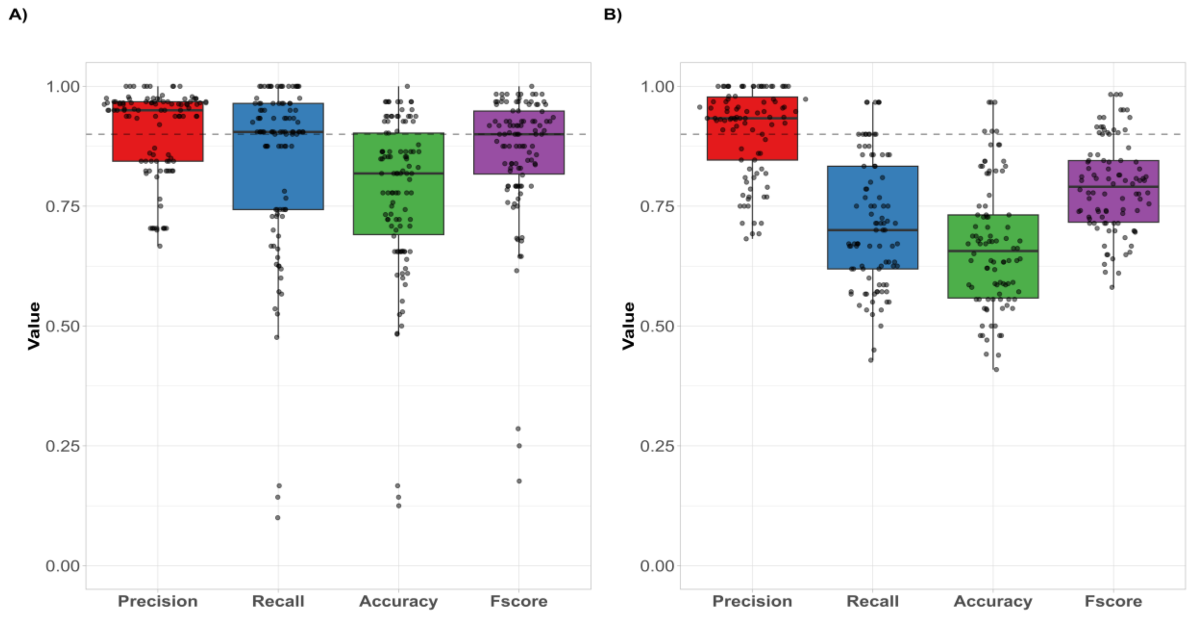

Supplementary Figure S4: PathoSeq-QC *in-silico* shotgun NGS performances. A grey dashed line marks the 0.9 value. **A)** PathoSeq-QC performance metrics on the shotgun dataset, with Precision, Recall, Accuracy, and Fscore mean values of 0.91, 0.85, 0.78, 0.86, and median values: 0.95, 0.91, 0.82, 0.90, respectively. These were evaluated considering only GATK variants passing default analyses thresholds. The reference set of variants for each sample was obtained from the SARS-CoV-2 database of variants, which is bounded with this distribution. Similar performances were observed using LoFreq results (data not shown, mean values: 0.90, 0.73, 0.67, 0.76, median values: 0.95, 0.90, 0.76, 0.86, respectively). **B)** PathoSeq-QC performance metrics on the amplicon-based dataset, with Precision, Recall, Accuracy, and Fscore mean values of 0.91, 0.71, 0.67, 0.79, and median values: 0.93, 0.70, 0.66, 0.79, respectively. These were evaluated following the same procedure used for the shotgun dataset.

S.5 PathoSeq-QC comparison with V-pipe

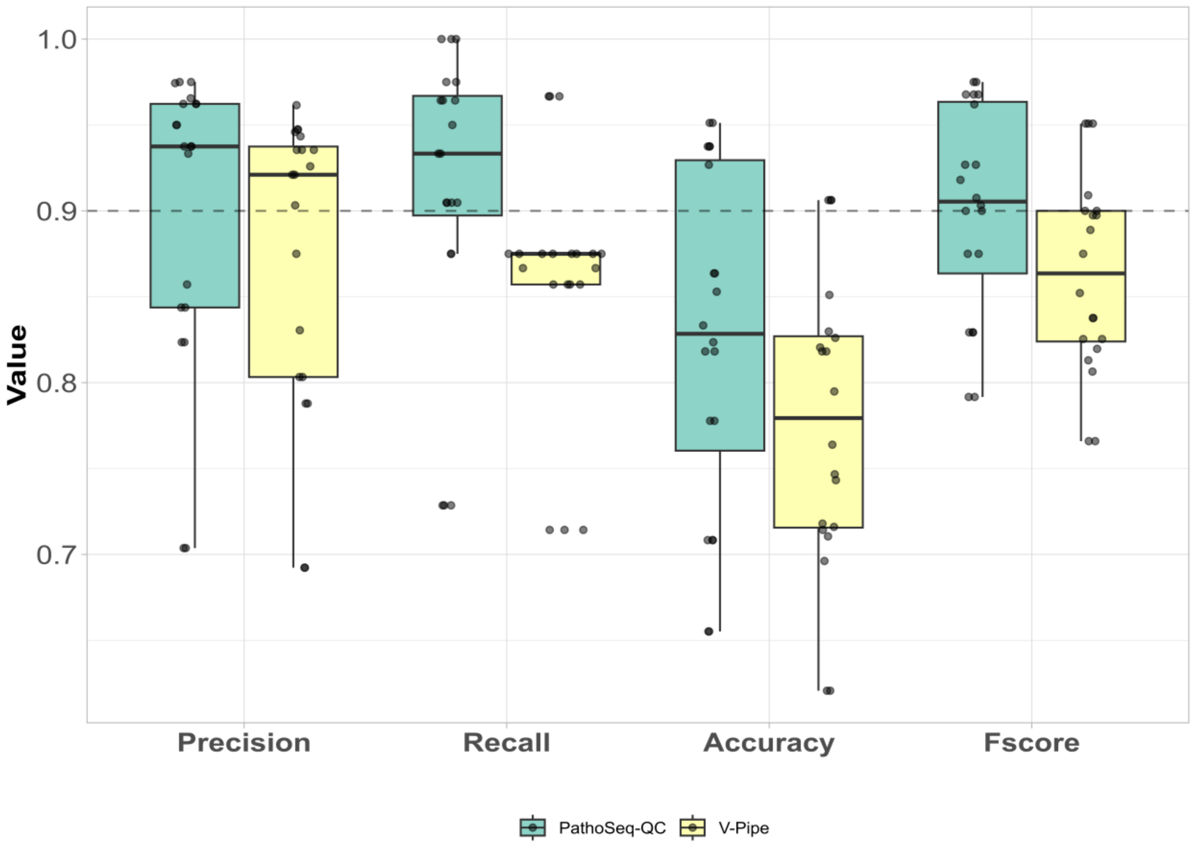

Supplementary Figure S5: Performance comparison between PathoSeq-QC and V-pipe 3.0 (v.7.32.4) [Fuhrmann *et al.*, 2024]. Due to the ability of V-pipe in analysing high coverage data, only > 1000x *in-silico* shotgun NGS raw reads were taken into consideration. Both tools were run with default parameters. No statistically significant differences were observed when comparing tool performances.

S.6 PathoSeq-QC species flexibility

A)

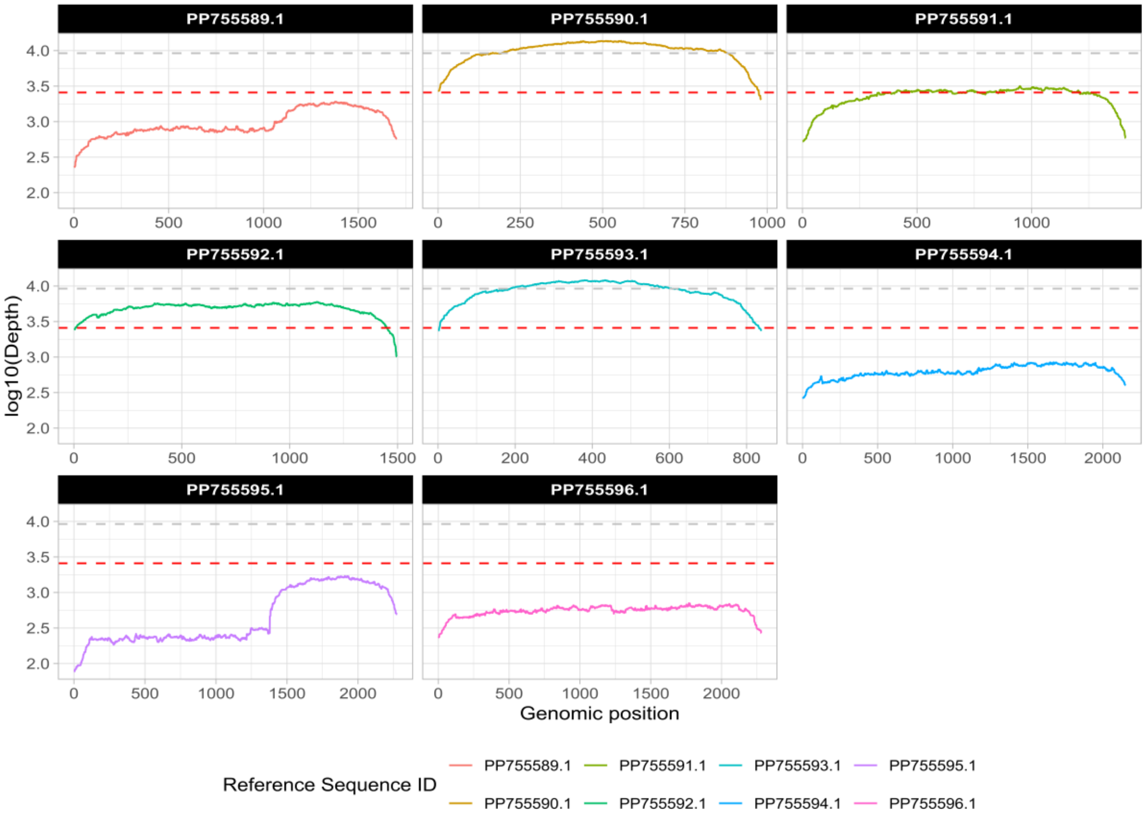

B)

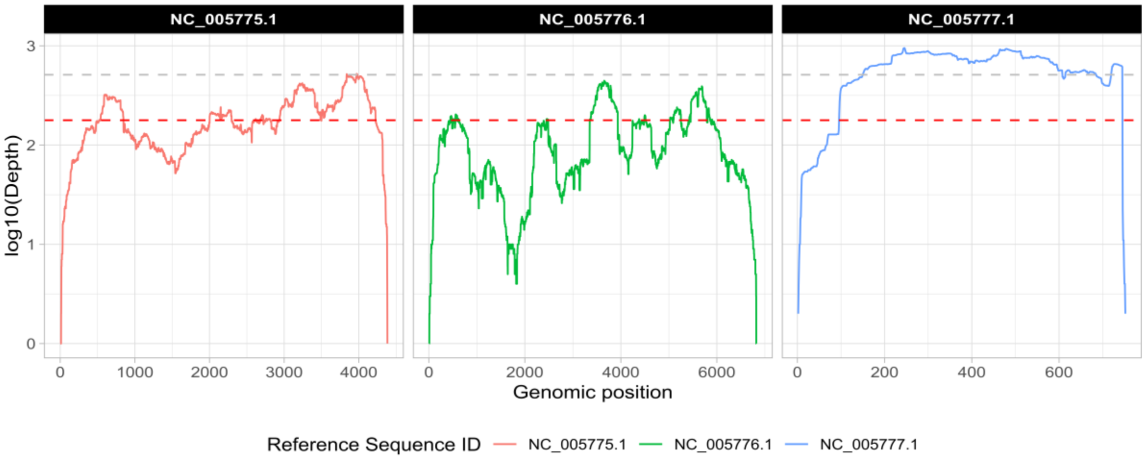

Supplementary Figure S6: Visualisation examples from the analyses of A(H5N1) and Oropouche viruses. PathoSeq-QC has been successfully applied with default filtering parameters on A(H5N1) (*--H5N1* option triggered) and Oropouche virus samples to prove it versatility across different viral species. **A)** Coverage distributions from the analyses of A(H5N1) virus raw data associated to run ID: SRR29851702. **B)** Coverage distributions from the analyses of Oropouche virus raw data associated to run ID: SRR14711849. Coverage distributions are evaluated after the mapping steps and reported for each reference genomic sequence (IDs of the sequences are shown). Mean coverage value per sample is visualised as a dashed red line, while a 2-fold standard deviation coverage value is displayed as a grey dashed line. Full analyses data and results can be found at the following DOI: 10.2905/572e9c76-be0d-4d36-977e-32ec063b93bb.

S.7 Coverage analyses on the representative sequence dataset

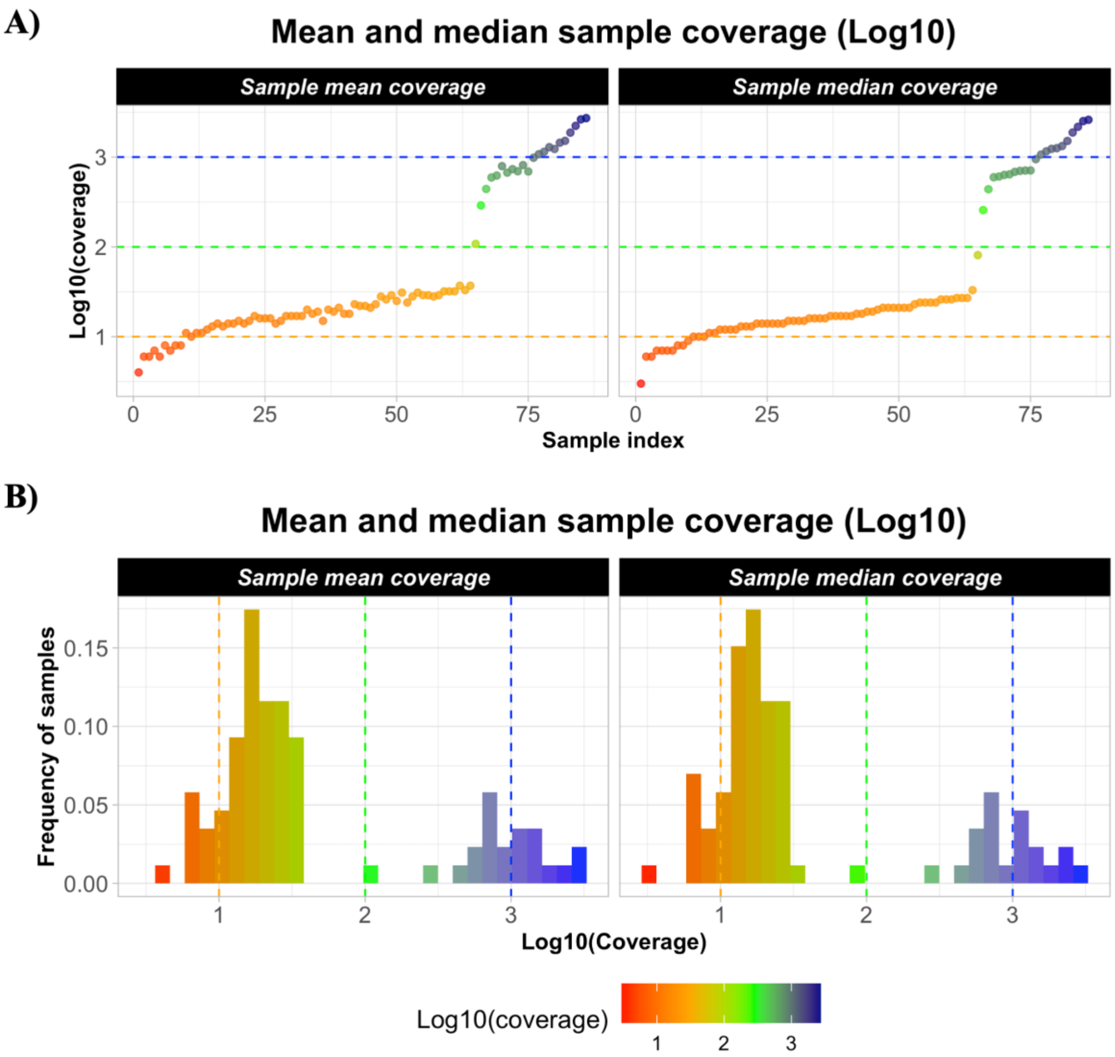

Supplementary Figure S7. Results of the coverage analyses on representative SARS-CoV-2 sequences displayed in logarithm scale. **A)** Scatterplot showing the mean and median coverage values per sample; **B)** Frequency of samples according to coverage values. These results highlighted how most of the samples were sequenced with low depth of coverage (< 50).

S.8 Heterogeneity analyses

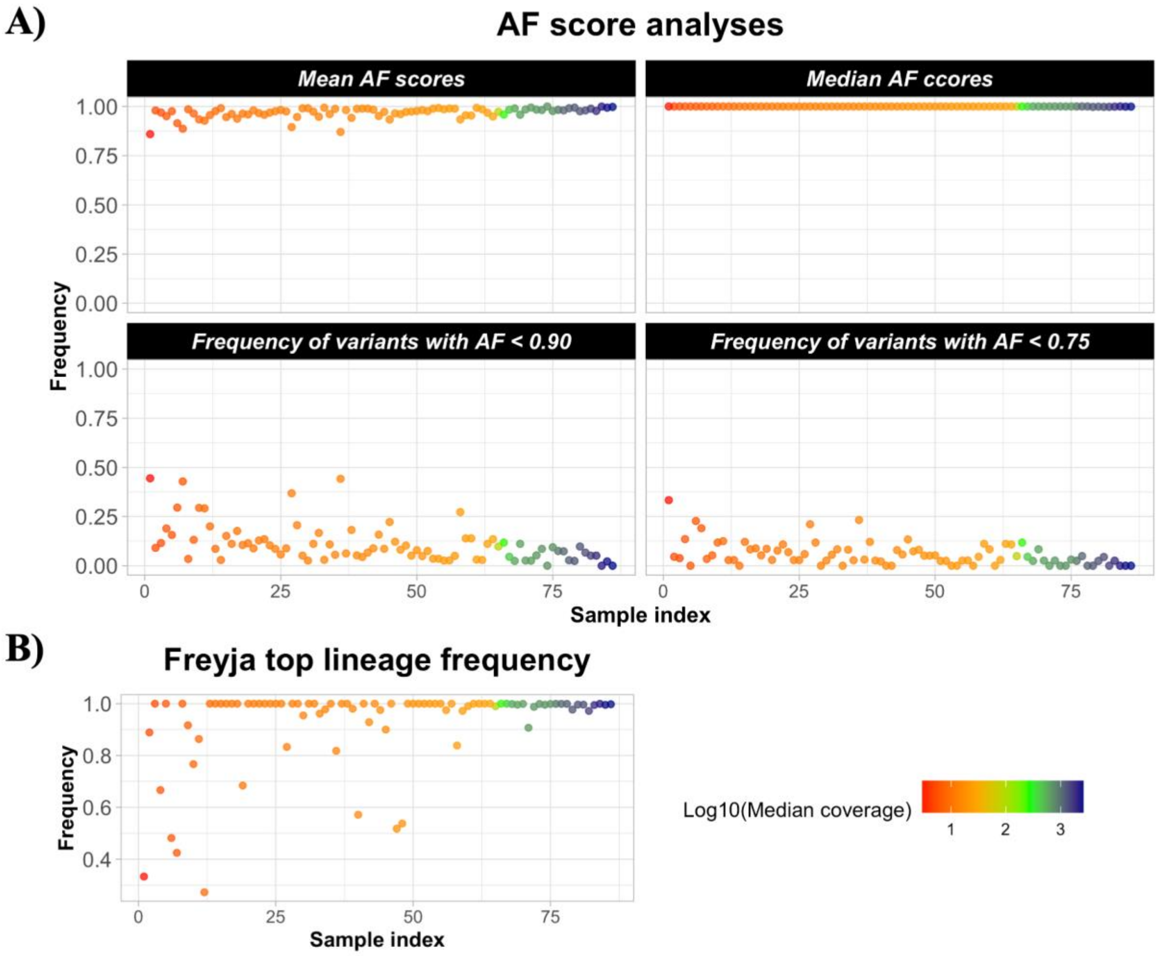

Supplementary Figure S8: Genomic heterogeneity of representative sequences. **A)** Results of the AF analyses. Mean and Median AF scores are reported for each sample. Additionally, for each sample, the percentage of variants showing AF values below 0.9 and 0.75 is displayed as it is an indicator of high genomic heterogeneity. **B)** Results of the *Freyja* analyses. The frequency of the top lineage identified by *Freyja* in the sample is reported. Both analyses underlined how samples sequenced with low coverage values were the most affected by potential high genomic heterogeneity.

S.9 Impact of Deduplication on Variant Support for raw data run ID: ERR7541889

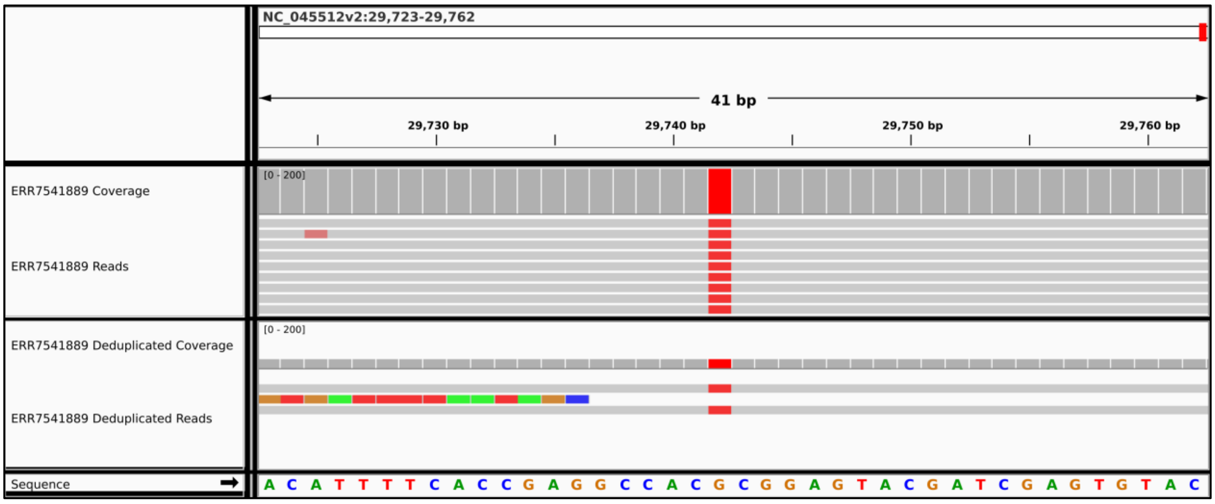

Supplementary Figure S9. Impact of Deduplication on Variant Support for raw data run ID: ERR7541889. *Integrative Genomic Viewer* (IGV) screenshots illustrating variant 29742:G-T (highlighted in red) in run ID ERR7541889. The removal of 169 duplicated reads significantly influences variant support. Middle panel shows the whole set of reads mapped to the variant locus. Bottom panel shows the retained reads after deduplication. Coverage tracks are depicted on a log scale and allow to compare the level of supporting reads.

S.10 Supplementary tables

Supplementary Table S1. Allele frequency score of variants detected by *GATK HaplotypeCaller* for samples run ids ERR6136423, ERR6187498 and ERR7541889. A single variant was identified and removed by our internal quality controls based on coverage values (highlighted in orange). POS: Genomic position, REF: Reference allele, ALT: Alternative allele, AF: Alternative allele frequency, DP: Depth, FILTER: Coverage filter outcome.

CHROM	POS	REF	ALT	AF	DP	SAMPLE	TOOL	FILTER
MN908947.3	14882	A	C	0.531	49	ERR6136423	GATK	PASS
MN908947.3	19220	C	T	0.553	47	ERR6136423	GATK	PASS
MN908947.3	24410	G	A	0.606	33	ERR6136423	GATK	PASS
MN908947.3	1878	C	T	0.52	25	ERR6187498	GATK	PASS
MN908947.3	19220	C	T	0.727	33	ERR6187498	GATK	PASS
MN908947.3	22028	GAGTTCA	G	0.889	9	ERR6187498	GATK	PASS
MN908947.3	22995	C	A	0.857	14	ERR6187498	GATK	PASS
MN908947.3	15521	T	A	0.65	20	ERR7541889	GATK	PASS
MN908947.3	19220	C	T	0.895	19	ERR7541889	GATK	PASS
MN908947.3	29742	G	T	1	2	ERR7541889	GATK	NA

Supplementary Table S2. *iVar* analyses results for variants not detected in samples ERR6136423, ERR6187498 and ERR7541889 by *GATK*. Variants at positions 21987, 24410 and 29724 were observed (highlighted in orange) but considered false positive calls by the tool due to the low number of supporting reads. Other variants were found at low AF values (< 0.5), indicating high genomic heterogeneity of the samples. Column description: POS: Genomic position, REF: Reference allele, ALT: Alternative allele, ALT_FREQ: Alternative allele frequency, TOTAL_DP: Total depth, PVAL: p value, PASS: Quality filter outcome.

SAMPLE	REGION	POS	REF	ALT	ALT_FREQ	TOTAL_DP	PVAL	PASS
ERR6136423	MN908947.3	21987	G	A	0.0869565	23	0.255556	FALSE
ERR6187498	MN908947.3	21987	G	A	0.0909091	11	0.52381	FALSE
ERR6187498	MN908947.3	24410	G	A	0.372093	43	3.3703e-06	TRUE
ERR6187498	MN908947.3	24410	G	+A	0.0232558	43	0.4	FALSE
ERR6187498	MN908947.3	28461	A	G	0.213235	136	3.92024e-10	TRUE
ERR7541889	MN908947.3	8835	T	C	0.538462	39	1.56026e-08	TRUE
ERR7541889	MN908947.3	25350	C	T	0.428571	35	5.72953e-06	TRUE
ERR7541889	MN908947.3	29742	G	T	1	2	0.333333	FALSE

References

Fuhrmann,L. *et al.* (2024) V-pipe 3.0: a sustainable pipeline for within-sample viral genetic diversity estimation. *GigaScience*, **13**, giae065.
